# Circulation of ideas about schizophrenia within the psychiatric context of Rio de Janeiro in the 1910s and 1920s

**DOI:** 10.1590/S0104-59702024000100057en

**Published:** 2024-11-04

**Authors:** Ana Teresa A. Venancio, Renilson Beraldo

**Affiliations:** i Researcher, Graduate Program in the History of Science and Health/Casa de Oswaldo Cruz/Fiocruz. Rio de Janeiro – RJ – Brazil ana-teresa.venancio@fiocruz.br; ii Doctor of the History of Science and Health, Casa de Oswaldo Cruz/Fiocruz. Rio de Janeiro – RJ – Brazil beraldo_rfs@hotmail.com

**Keywords:** Schizophrenia, Paul Eugen Bleuler (1857-1939, Ernst Kretschmer (1888-1964, Rio de Janeiro, Circulation of ideas

## Abstract

The article examines the circulation of the schizophrenia category in debates and psychiatric spaces in Rio de Janeiro at the beginning of the twentieth century. It analyzes this category in the scientific exchange between Eugen Bleuler and Ernst Kretschmer – observing correspondences between psychodynamic and constitutionalist theories in the European context – and then pursues its use in the Observation Pavilion of the Hospício Nacional de Alienados, in articles and scientific meetings. We investigated how its circulation was simultaneous in scientific and assistance spaces. Furthermore, we also demonstrate that, as occurred in the European context, Brazilian psychiatrists actively participated in the transnational circulation of the schizophrenia category through its appropriation and expanded use of the semantic field “schizo” according to local interests.

Since its establishment as a medical specialty at the dawn of the nineteenth century, psychiatry has addressed the problem of the body/mind relationship in defining mental pathologies. Its subsequent developments led to the formation of a set of discourses and theories which, in each historical period and place, has sought to define what should be seen as a phenomenon relative to mental pathology: a pathology that is simultaneously physical and moral, different from those that are specifically organic but for which substantiation is constantly sought in the physical body. Because of the specific nature of the pathology it addresses, psychiatry is not like other medical specialties. The tension or oscillation between psychiatric ideas, theories, or therapies which are more concerned with the physical dimension of the human being and others that favor analysis of the moral or psychological dimension (this latter in contrast to the bodily dimension) has been a constant in the history of psychiatry, both diachronically and synchronically.

Starting from this perspective about the object of psychiatry, here we present how schizophrenia as a category – which was coined by the Swiss psychiatrist Paul Eugen Bleuler (1857-1939) (Bleuler, 1911) – circulated within the context of Rio de Janeiro, Brazil, translating ideas related to the modern cultural ability to consider the existence of an individual who is split within himself. Here we follow the international historiography^1^ that affirms the importance of the relationship between psychiatry and psychoanalysis in creating this diagnosis, based on the idea of a dynamic psychological makeup, but we also highlight the contribution of theories related to corporality in its anatomic/organic dimension to the affirmation and circulation of schizophrenia in Brazilian psychiatry, similar to what occurred in other countries. Through debates around schizophrenia, we observe the dissemination and circulation of ideas from psychoanalysis and its emphasis on the psychological dimension of individuals, associated with the constitutionalist theories of the psychiatrist Ernst Kretschmer (1888-1964).

From our perspective, this apparent duality of viewpoints expresses a correlation between psychodynamic and constitutionalist theories precisely because the latter favored addressing the mind/body relationship. Starting from the notion of individual totality, based on medical holism and the concepts of organic unity and physical/moral unity, Kretschmer’s constitutionalist theories included a look at and interest in the very psychological dimension required in the debate on so-called mental pathologies. In this sense, we observe that the moral psychological dimension of this new category, “schizophrenia,” with its emphasis on the dynamism of the psyche, was simultaneously read and disseminated from a holistic vision of the body.

Our objective, therefore, is to reveal how the diagnosis of schizophrenia (coined by Bleuler) and expression of psychodynamic theories circulated and was appropriated within the field of psychiatry in the city of Rio de Janeiro via scientific debate and care practices at two institutions that were important at that time. First, the Brazilian Society for Psychiatry, Neurology, and Legal Medicine [Sociedade Brasileira de Psiquiatria, Neurologia e Medicina Legal, SBPNML], through its scientific journal *Arquivos Brasileiros de Neuriatria e Psiquiatria*,^2^ Brazil’s first publication specialized in the area of psychiatry; the second institution is the Observation Pavilion at the National Asylum for the Insane [Hospício Nacional de Alienados, HNA],^3^ which from the early 1890s served as both the entry point for care and a space for teaching and research in clinical psychiatry for the Rio de Janeiro School of Medicine [Faculdade de Medicina do Rio de Janeiro, FMRJ].

In this article we concur with Secord (2004, p.661-662) that knowledge can be observed in the form of communicative actions. In other words, in this case scientific discussions (meetings, conferences, articles) and care practices are understood as acts of scientific communication. These actions in turn, according to Raj (2013, p.344), help build spaces for circulation. In this way, in dialog with these authors, we understand that circulation of knowledge results from contact between people, institutions, and ideas. This contact not only helped to structure diagnostic categories, but specifically by examining the dialog between Bleuler and Kretschmer, this structuring overcame national and linguistic boundaries to inform a transnational circulation of knowledge (Weinstein, 2013, p.17). We consequently do not view the role of isolated actors as points of origin or potential consumers of theories or concepts; instead, we are visualizing the contexts and spaces for circulation and appropriation of the semantic domain related to schizophrenia that are in line with specific interests and intellectual configurations (Patiniotis, 2013, p.773-774).

## Dialogs between Bleuler and Kretschmer in the circulation of schizophrenia as a category

When he created the category of schizophrenia, the Swiss psychiatrist Eugen Bleuler was director of the Burghölzli Sanitarium (Zurich’s university hospital), where he worked most closely with Carl Gustav Jung (1875-1961), who was a young man at that time and introduced Bleuler to the work of Sigmund Freud. It was in this setting, at the intersection between psychoanalysis and psychiatry in the early twentieth century, where a “psychodynamic psychiatry” was established and the new category emerged. Bleuler’s intention was to replace the diagnosis of dementia praecox, which was created by the German psychiatrist Émil Kraepelin (1856-1925) in the late nineteenth century and widely used in the northern and southern hemispheres. According to the historiography, the category of schizophrenia emerged in 1911 when Bleuler published his seminal book *Dementia praecox or The group of schizophrenias.* He defined it based on four fundamental symptoms: disturbed thinking, affective abnormalities, ambivalence, and autistic withdrawal. Bleuler himself coined this last term to describe an escape from reality. From then onward, he circulated this new category with his publications, such as his 1917 treatise *Lehrbuch der Psychiatrie,* and in presentations at congresses such as “La schizophrénie” at the Congrès des Médecins Alienistes et Neurologistes de France et des Pays de Langue Française in Geneva and Lucerne in 1926, where the French psychiatrist and neurologist Henri Claude (1869-1945) also presented his work on the topic. Bleuler adhered to some basic principles of psychodynamic psychiatry, such as the idea of a psyche in movement which also acted through its unconscious dimension, but still had reservations about Freudian ideals related to the centrality of sexuality and its significance in psychiatric processes.

Ten years after Bleuler’s book, Kretschmer published the first edition of one of his most widely translated works, *Körperbau und Charakter* [Physique and Character], in 1921. Starting from two classic mental pathologies created by Kraepelin (dementia praecox and manic-depressive psychosis), Kretschmer correlated bodily elements related to anatomy, physiology, metabolism, and endocrinology with characteristics of temperament, such as cyclothymia and schizothymia, cycloidia and schizoidia. In this way he established a dual constitutional typology in which the morphological and temperament-related characteristics of manic-depressive and schizophrenic individuals could be found in healthy individuals in an “attenuated” stage (Bercherie, 1980, p.159-168). In doing so, Kretschmer established two important analytical points: he provided a theoretical instrument to analyze the moral/physical correlation, and relativized the divisions between psychopathological phenomena and mental states considered healthy.

Although he based his initial research on Kraepelin’s categories, Kretschmer was harshly criticized by the members of the German Institute of Psychiatric Research in Munich [Deutschen Forschungsanstalt für Psychiatrie], including Kraepelin himself (Engstrom, 2008, p.241). They accused him of being very “psychological” and not utilizing the “biological” in his observations, and also defended the end of strict separation between endogenous and psychogenous causes (Priwitzer, 2007, p.281-282).

In the case of schizophrenia, Kretschmer’s dialog was closer to that of Bleuler, who, in turn, did not hide his admiration for Kretschmer since he was a disciple of Robert Gaupp (1870-1953) in Tübingen in mid-1918 (Beraldo, 2021, p.179). According to Patrício (2001, p.86-88), Kretschmer learned the character approach from Gaupp, in which one could look for the early manifestations of an illness in an individual’s personality traits. His use of this approach was important, since it favored his descriptions of abnormal schizoid and cycloid personalities correlated with schizophrenia and manic-depressive psychosis, respectively, as attenuated forms of these conditions.

Kretschmer maintained that a diagnostic approach should encompass the entirety of an individual’s psychophysical dimension, which was thought to be comprised of three aspects. The first was “constitution,” which expressed the confluence between the somatic and the psyche. In this sense, Kretschmer’s constitutional types speak to the relationship between body, temperament, and character: “temperament” was related to endocrine and neural physiology, while “character” addressed the psychological dimension of the individual, formed by experiences prior to the emergence of the psychosis. These experiences could then function as “key situations” responsible for the appearance of the pathology. For Kretschmer, body structure, figure, or physique (bodily properties) were considered expressions/reactions of the individual constitution which had a hereditary basis (Beraldo, 2021, p.177-179). From this perspective, Kretschmer’s research agenda investigated the frequency with which certain psychiatric forms (schizophrenia and manic-depressive psychosis) could be correlated to certain types of body structures.

Kretschmer (1923) also continued to debate the seat or residence of the spirit or soul, expanding on his reflections published since 1920 in his text *Medizinische Psychologie* [Medical Psychology] (Kretschmer, 1957). Based on the work of neovitalist authors such as the philosopher and biologist Hans Driesch (1867-1941) and psychiatric contributions like those of Bleuler, he believed the psyche was not linked to the exclusive functions of any specific organ (like the brain or brainstem) but instead was seated anatomically as well as physiologically, considering the body in its entirety (Kretschmer, 1957, p.11-12). This integral vision precedes Kretschmer’s best-known book (1921). His interest in the moral and psychological dimension also was expressed by his use of notions like character traits and reaction types, based on the development of the multidimensional diagnostic technique [Mehrdimensionalen Diagnostik] (Engstrom, 2008; Priwitzer, 2007).

From the historiography and primary sources, we can observe that Bleuler and Kretschmer maintained intellectual exchanges and referenced each other since after First World War. Kretschmer began publishing around 1914 on topics such as deliria and hysteria (1914-1917), moving on to analyze the concept of the unconscious (1919) in which he dialogued with Bleuler, culminating in his 1918 dissertation on paranoia and character theory, as well as his 1921 book on the constitution (Priwitzer, 2007, p.289-292). Meanwhile, Bleuler read Kretschmer’s 1918 psychiatry dissertation entitled *Der sensitive Beziehungswahn* [Reference-Sensitive Delirium], which examined the correlation between previous temperament, experience, and life situations in determining psychosis. But the two only met in 1920, at the 59th Meeting of the Swiss Psychiatry Association (Priwitzer, 2007, p.75-76).

Bleuler (1921) was also one of the first specialist readers to summarize *Körperbau und Charakter*, in the same year this text was published. In 1922, in the preface to the second edition of his own book, Kretschmer stated that the facts he had observed in 1921 had already been partially known to Bleuler for quite some time (Kretschmer, 1925, p.13). In fact, Kretschmer reinforced what R. Gaupp had written in 1921 in the preface to the first edition of the book, in other words, what had been intermediated by Bleuler and made “deeper understanding of the symptoms and individual content (character and history of the pre-psychotic personality) in schizophrenic processes” viable in psychiatry (Kretschmer, 1925, p.10).^4^ According to Kretschmer himself, the pathway to psychopathological examination in the areas that form the line between psychopathy and normality (which are expressed in schizoid disorders) had been first established by Bleuler (Kretschmer, 1925, p.122).

Other exchanges between the Tübingen and Zurich schools appeared in the third (1920) and fourth editions (1923) of Bleuler’s *Lehrbuch der Psychiatrie*. In the former, Bleuler (1920, p.403) used Kretschmer’s 1918 thesis to talk about “primitive reactions” [Primitivreaktionen] as exaggerated character traits (shouting, tantrums, stupor, depression). In the latter, Bleuler expanded the section entitled “Classification of mental illnesses” to include some of Kretschmer’s formulations presented in his 1921 book. According to Kretschmer, the role of character dispositions and constitutions (manias involving persecution and grandiosity, for example) as predisposing for deliria and illnesses is highlighted, ultimately producing extensive commentary about Kretschmer’s multidimensional diagnostic and his contribution to the study of temperament (Bleuler, 1923, p.132-134). Kretschmer, in turn, borrowed two terms from Bleuler: autism, characterized as “living within oneself” (Kretschmer, 1925, p.147), and ambivalence, namely “leaping forward and backward between emotion and willingness” (p.177). These terms were mobilized by Kretschmer to characterize the schizoid temperament in correlation with the asthenic and athletic physiques.

The circulation of psychiatric ideas between Bleuler and Kretschmer which we are discussing here, in their cross-referencing in the literature and in their meeting at the congress, also took place as both authors appropriated notions from the work of other psychiatrists who were widely cited within the Brazilian context, such as the French psychiatrist of Polish origin Eugène Minkowski (1885-1972) and his wife, the psychiatrist Françoise Minkowska-Brokman (1882-1950).^5^ Both read Kretschmer and assisted Bleuler at Burghölzli, the university clinic. After First World War, when they were established in Paris, Minkowska conducted research that reconstructed the family trees of workers diagnosed as epileptics and schizophrenics. With information obtained from living relatives, she described the epileptoid or glischroide constitution (Yahn, 1952, p.93-94).

Minkowski resumed his medical studies and also worked at the Hospital St. Anne in Paris. In 1925, he published “La genèse de la notion de schizophrénie et ses caracteres essentiels” in *Evolution Psychiatrie*, and the following year, defended his doctoral thesis in medicine entitled *La notion de perte de contact avec la réalité et ses applications en psychopathologie*. This work was strongly influenced by the theories of Henri Bergson and conducted under the supervision of Henri Claude, a psychiatrist whose ideas were also present in the circulation and appropriation about schizophrenia within Brazil’s specialist scientific context. Minkowski’s research maintained that psychiatric investigations related to the constitution should focus on the patient’s past to find the essential traits of the current psychosis in specific pre-existing details of the character (in other words, the psychological dimension of the individual, comprised of experiences that took place prior to the psychopathology), as Bleuler and Kretschmer had observed. Such rejoinders can be seen in *La schizophrénie* (Minkowski, 1927), for example. According to Silva Neto (2004, p.51-54), with regard to the Bleulerian idea of schizophrenia, Minkowski highlighted the notion of “loss of vital contact with reality,” emphasizing concepts like duration and time to discuss the nuances of the schizophrenic experience. To Minkowski, this notion became the point where the Zurich school (Bleuler) intersected with the ideas of Bergson (Minkowski, 2019, p.59; originally published in 1926). What we can confirm is that the record of this transnational circulation of ideas and people, as well as the reformulation of the concepts, signals a viewpoint that is located far from the appropriations of Kretschmer’s work in the 1930s, where the constitutional types come to be interpreted as signs of physique that reveal indelible traits related to an individual’s temperament and character as part of a eugenic worldview.

## Circulation of the category of schizophrenia in psychiatry in 1920s Rio de Janeiro

The category of schizophrenia circulated more significantly within the Brazilian psychiatric context from the second half of the 1920s onward; as we show in this original work, it took place though the knowledge disseminated at the country’s main insane asylum as well as in scientific debates, expressed in academic dissertations, articles in specialized periodicals, congresses and minutes of scientific societies dedicated to psychiatric topics and to the young field of neurology. To do so, we start from the historiography which observes that appropriation of this category primarily took place via psychiatry, although we found schizophrenia and related terms later in work related to neurology.

With regard to neurology, it is notable that in 1918, the neurologist Antônio Austregésilo (1876-1960) referred only to dementia praecox (and not schizophrenia) in his article for the Brazilian medical journal *A Patologia Geral: Revista de Medicina e Ciências Afins*, when discussing the diagnosis of cataphrenia he had created. This article was published the following year in French in the *Revue Neurologique* and presented years later, in March 1926, in a session of the Paris Psychiatric Society, which he belonged to. But in 1918, Austregésilo was more active in discussions around the category of dementia praecox and how it determined intellectual deterioration. Although his new diagnosis, cataphrenia, mostly centered on the areas between psychopathological events and healthy states, only during his 1926 presentation did he utilize terms related to schizophrenia in reference to concepts such as schizothymia and schizoid, which followed Kretschmer’s example and reinforced the relativization between healthy and pathological states (Beraldo, 2021, p.229-233).

The category of cataphrenia, which was in line with Kretschmer’s constitutionalist theories, was produced via scientific interests within the local context of Rio de Janeiro. On the one hand, it met the needs of the first head of the neurology department in the Rio de Janeiro School of Medicine to align the institution’s discussions on clinical practice and general pathology with respect to constitutionalism, strengthening a holistic view of medicine defended by some well-known professors who considered the growing specialization and fragmentation of medicine at that time to be harmful (Beraldo, 2021, p.95-139). It does not seem a casual coincidence that Austregésilo’s first text (1918) was published in the medical journal *Patologia Geral*. On the other hand, the original creation of the cataphrenia as a category also served to highlight the prominent position new specialty of neurology had achieved in regard to current psychiatric knowledge in Rio de Janeiro. Although these medical specialties remained together in the SBPNML, we also note that they competed at that time and separate spaces were required for their discussions, such as the different commissions created to specifically address these topics (Cerqueira, 2014). It was through these routes that the sciences related to mental aspects specialized and gradually differentiated themselves in the local context. In this way, the chair of neurology only utilized terminology related to schizophrenia when this category was already circulating more broadly in the specialist milieu of psychiatry in Rio de Janeiro, where it was first “received.”

With regard to the pioneering nature of psychiatry in the circulation of schizophrenia as a category in Brazil, Murillo de Souza Campos (1887-1968) confirmed at that time that Hermelino Lopes Rodrigues (1899-1971) had authored the first work produced in the country on schizophrenia (Campos, 1929, p.178), affirming its use in the field of psychiatry. Silveira (2009) also states that the idea of schizophrenia was introduced by the psychiatrist Lopes Rodrigues in 1926, with his defense of the dissertations “Etiopatogenia da demência precoce” [Etiopathogenesis of Dementia Praecox] and “Estudo clínico das esquizofrenias” [Clinical Study of the Schizophrenias] as part of his application process for the position of full professor of psychiatry at the Federal University of Minas Gerais School of Medicine (which he obtained). According to Silveira (2009), Lopes Rodrigues began his course in medicine at the Bahia Medical School in 1916 and four years later transferred to Rio de Janeiro, where he completed his studies, simultaneously studying under Henrique Britto de Belford Roxo (1877-1969) and working with Juliano Moreira (1873-1933) at the National Asylum for the Insane. In this psychiatric context, Rodrigues would have known about and been impressed by the “psychological aspects” covered in Bleuler’s diagnosis (Silveira, 2009, p.589).

Meanwhile, Venancio (2010) demonstrates that a debate existed on the uses of the categories of dementia praecox and schizophrenia in 1929 between the already well-established Henrique Roxo (a full professor at the Rio medical school and director of the Observation Pavilion at what was then the National Hospital for the Insane) and Murillo de Campos. The two psychiatrists shared their work at the third Brazilian Congress of Neurology, Psychology, and Legal Medicine in Rio de Janeiro, and that same year published them in the *Arquivos Brasileiros de Neuriatria e Psiquiatria* (Roxo, 1929; Campos, 1929). Venancio demonstrates how this process of assigning and scientifically defining the diagnoses of dementia praecox and schizophrenia connected to determining the difference between what should and should not be seen as phenomena related to mental pathology. On one hand, Henrique Roxo adhered to a static vision of illness, favoring Kraepelin’s notion of dementia praecox in which physical and organic characteristics were more decisive for qualification of the pathology and led to complete deterioration of the individual. Meanwhile, Murillo de Campos emphasized the psychodynamic nature of schizophrenic morbidity, discussing the psychological aspects which, as he saw it, were more relevant to the definition of this mental pathology (Venancio, 2010, p.341).

None of the authors cited, however, invested in the joint analysis of different actors, processes, and spaces of science and care where the diagnosis of schizophrenia circulated and how this event was simultaneously informed by Bleuler’s and Kretschmer’s theories. For this reason, here our goal is to demonstrate the processual way in which the interest in and presence of this category grew from the mid-1920s in the two main spaces where psychiatry was institutionalized in Rio de Janeiro at that time: the Observation Pavilion at the National Asylum for the Insane, and the SBPNML with what at that time was the *Arquivos Brasileiros de Neuriatria e Psiquiatria*, a Brazilian periodical specialized in psychiatry which published not only scientific articles but also the minutes of this society and discussions of clinical cases that were presented at the meetings of this group.

The Observation Pavilion was not only where triage for psychiatric care took place (regulations considered a patient would stay for a period of up to 15 days), but also, as mentioned earlier, was where students interested in the subject would undergo their specific training, attending classes and presenting clinical cases.^6^ Quantitative mapping^7^ of the diagnoses made in men and women admitted to the Observation Pavilion at the HNA between 1903 and 1930 shows a higher overall number of men (23,451) than women (15,019), totaling 38,470 admissions^8^ to the Pavilion, with similar distribution of male and female admissions during this period. Of this total of admissions, we can also see that schizophrenia was utilized as a diagnosis since 1926, in men as well as women, at the same time that the number of patients considered to have dementia praecox decreased.

**Figure 1:**
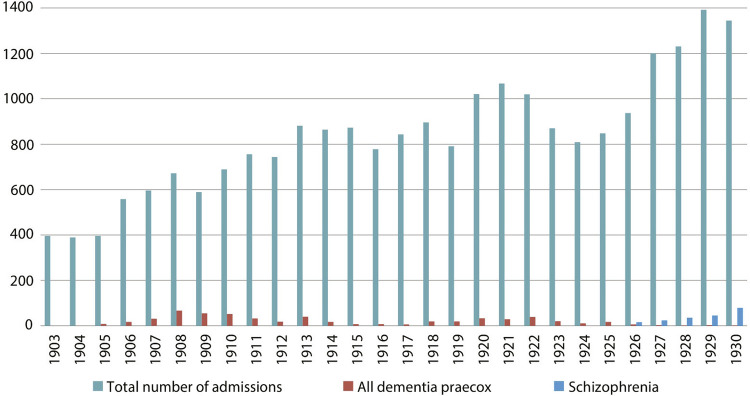
: Graph of diagnoses of dementia praecox and schizophrenia in relation to total number of admissions to the Observation Pavilion at the Hospital Nacional de Alienados, men, 1903-1930 (Source: register books from the Observation Pavilion at the HNA within the collection of the Institute of Psychiatry at the Federal University of Rio de Janeiro)

**Figure 2:**
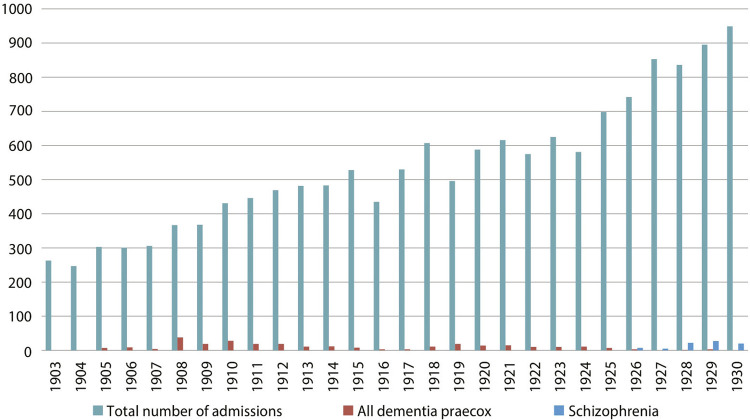
: Graph of diagnoses of dementia praecox and schizophrenia in relation to total number of admissions to the Observation Pavilion at the Hospital Nacional de Alienados, women, 1903-1930 (Source: register books from the Observation Pavilion at the HNA within the collection of the Institute of Psychiatry at the Federal University of Rio de Janeiro)

And how were these diagnostics presented by psychiatric science in Rio de Janeiro? The scientific articles and other published texts (some reviews, one bibliographical note, and a bulletin about the scientific institution), as well as the clinical discussions at SBPNML which were also publicized in the *Arquivos Brasileiros de Neuriatria e Psiquiatria*, agree in terms of the similar movement through which the diagnosis of schizophrenia began to be used at the Observation Pavilion at the Hospital Nacional, as publications on this topic began to increase in number from the 1920s. As we know, many of the psychiatrists who wrote on this topic were also physicians at the National Hospital and its Observation Pavilion, using the clinical cases for their research and dissertations.

From the 1920s, we can observe that the category of schizophrenia assumed a larger presence compared to dementia praecox, although references to the latter did not disappear completely. In 1905, the first year that the *Arquivos Brasileiros* was published, this journal contained an article discussing dementia praecox. During the 1900s and 1910s we have 23 texts discussing the diagnosis of dementia praecox, with no reference to the category of schizophrenia, as this only appeared in Europe the following year. These texts relate the Kraepelinian category to various pathologies (syphilis, tuberculosis, paranoid dementia, and paraphrenia), but principally to manic-depressive insanity, thus pursuing one of the canons of psychiatry at that time coined by Kraepelin, namely the distinction between dementia praecox and manic-depressive psychosis (Kendler, Engstrom, 2018).

The first reference to Eugen Bleuler appears in an article by Waldemar de Almeida (1917). Almeida received his degree from the Rio de Janeiro Medical School in 1909, but since the previous year had served first as an medical intern in the Observation Pavilion and then as an assistant and physician providing care at the HNA until 1922, when he became the director of the Asylum Colony at Vargem Alegre, while simultaneously maintaining his own private practice from 1915 to 1922 (Cerqueira, 2014, p.30). In his text, which discussed diagnosis, remissions, and treatments for patients with dementia praecox, Almeida mentioned the controversy between Bleuler and Kraepelin, defending the ideas of the latter. Almeida’s text presents what can be seen in the diagnostic debates of the 1910s, when critiques of the breadth of Bleuler’s idea prevailed; this is exemplified in the citation below by Austregésilo (1918), as the diagnosis of dementia praecox solidified, as Almeida (1917, p.73) summarized in his own text: “In this work, after highlighting the role of dementia praecox created by Kraepelin, the author recalls a brief summary of its critics, supporters, and opposition as it came to be definitively established in mental pathology.” Also: “Bleuler’s concept surrounding schizophrenia is not yet well defined, because, looking again at the psychiatry studies in Zurich, I note that borders were greatly expanded, based more on studies in psychoanalysis than in the clinic per se” (Austregésilo, 1918, p.124).

During the 1920s, after First World War, ideas and actors circulated widely in the fields of psychiatry and neurology in Brazil, tightening ties with the Germanic context and resuming contacts with French physicians who worked with mental illness (Venancio, Cerqueira, 2016, p.13-15, 22) like the neurologist Joseph Babinski and psychologist George Dumas, the latter of whom had maintained cultural and scientific exchanges with Brazilian psychiatrists since before First World War. In the case of Germany, the scientific exchanges prior to the war when Germans reached out to Ibero-American countries expanded after the global conflict, as Brazilian physicians become increasingly interested in specialization courses at German university centers and psychiatric hospitals, inviting specialists to give lectures and teach courses in Brazil (Venancio, Cerqueira, 2016, p.22, 33; Muñoz, 2018, p.140-141).

Along with these scientific activities, the circulation of books and specialized articles can also be seen; we can note that many of the authors cited on topics related to psychiatry by physicians in Rio de Janeiro were French as well as German. Still, as noted by Beraldo (2023), there was also interchange between Spanish and Brazilian psychiatrists, as well as references to psychiatry in the Germanic context brought in by Spaniards. Murillo de Campos is an excellent example of this moving circuit of ideas related to schizophrenia. In his efforts to follow Bleuler’s ideas, Campos (1929) cited works by the Swiss psychiatrist published in several languages: the 1911 *Dementia precox oder Gruppe der Schizophrenien*; *Lehrbuch der Psychiatrie* from 1924; and “La schizophrénie,” presented at the Congrès des Médecins Alienistes et Neurologistes de France et des Pays de Langue Française in October 1926. He also in the same article utilized Kretschmer (in the original German) and Eugène Minkowski (in French) at the same time that he referenced other Germanic authors translated into Spanish, such as the *Tratado de las enfermedades mentales*, edited by Oswald Bumke (1877-1950) and published in Barcelona in 1926, and *La psiquiatria* by Hanz Walter Gruhle (1880-1958), simultaneously published in Barcelona and Buenos Aires in 1925.

From this perspective, the circulation of Bleuler and Kretschmer’s theories outside the Germanic context (through versions in other languages) catalyzed their use by diminishing linguistic distance, an important element for scientific exchange, as noted by Burke and Hsia (2009, p.13-17). Specifically with regard to Kretschmer, Beraldo (2021) indicates that the psychiatrist himself published a summary of his ideas in Spanish in the prestigious journal *Revista de Occidente* (1923). Founded by the Spanish essayist and philosopher José Ortega y Gasset (1883-1955) in Madrid in 1923, it circulated until the start of the Spanish Civil War, bringing together a select and diverse number of foreign collaborators like the physicist Albert Einstein, the philosopher Max Scheller, the sociologist Georg Simmel, the poet Pablo Neruda, as well as Spaniards such as the philosopher Fernando Vela (the cofounder of the journal), the endocrinologist Gregório Marañon, and the psychiatrist José Miguel Sacristán. The synthesis of Kretschmerian ideas therefore spread within a space for disseminating a variety of scientific topics, along with new developments in the areas of philosophy, literature, aesthetics, and texts related to the decay and environmental crisis present in Europe after First World War (Linacero, 2000, p.379).

It is here, in this zone where ideas, authors, and diagnostic debates circulated within the psychiatric context of Rio de Janeiro, where broader use of the category of schizophrenia can be seen throughout the 1920s. During this decade, the journal *Arquivos Brasileiros de Neuriatria e Psiquiatria* published six articles with the first references to schizophrenia. The first authors, Almeida (1920) and Conrado (1921), did not address it as a diagnostic category, but rather as a “form” or “symptom” that could exist in cases of cerebral syphilis. The text by Almeida (1920) “Sífilis cerebral de forma esquizofrênica” [Schizophrenic-form cerebral syphilis] was in fact a one-paragraph commentary on the text of the same name published in *Arquivos Rio-grandenses de Medicina* in January of that year. The next text (Conrado, 1921) also mentioned it as more of a type than a diagnosis: “Estudo clínico da forma esquizofrenoide da sífilis cerebral” [Clinical study on the schizophrenoid form of cerebral syphilis]. This work resulted from clinical observations in the Pinel Section of the Hospital Nacional de Alienados, which was led by Ulisses Viana (1880-1939). Since the 1910s, Vianna, his team, and their partners had been interested in research on syphilis, including how it was related to dementia praecox (SBPNML, 1914; Vianna, Moses, 1919). Since the early 1920s, references to schizophrenia (when they occurred) more resemble symptoms than a nosological entity, like dementia praecox occasionally appearing as a secondary topic related to a central discussion of other diagnoses which were the object of research at that time, such as “cerebral syphilis.”

After these articles in 1920 and 1921, in *Arquivos Brasileiros de Neuriatria e Psiquiatria* we encounter the publication of various minutes from ordinary sessions of the SBPNML in 1920, 1923, 1924, and 1926-1929 mentioning pathological studies that make reference to schizophrenia and related terms. The minutes actually related what the reporter selected from the in-person discussions on a variety of topics, including discussions on clinical cases that took place during the meetings of the society. In this sense, we cannot say that they reveal the entire content of the discussions that occurred among peers, although it is certainly significant that in the case in point, an increasing number of references to schizophrenia were the order of business in the field of psychiatry in Rio de Janeiro, in different types of texts, including debates on clinical cases mostly from the HNA: “Dr. Xavier de Oliveira presents two cases, one of schizophrenia and the other of dementia praecox. He briefly describes the schizophrenic constitution and ends by questioning whether ‘dementia praecox’ is the same thing as ‘schizophrenia’” (SBPNML, 1927, p.178-179; highlights in the original).

**Table 1:** : Frequency of texts in the *Arquivos Brasileiros de Neuriatria e Psiquiatria* on early dementia and schizophrenia

Period	Mainly related to dementia praecox	Mainly related to schizophrenia	Total number of texts and session minutes^*^
1905-1909	4 texts	-	4
1910-1919	7 texts; 12 sets of minutes from SBPNML meetings	1 article (1917)	7 texts + 12 sets of minutes = 19
1920-1929	3 articles; 6 sets of minutes	6 articles; 4 sets of minutes	7 texts + 10 sets of minutes = 17
1930-1939	2 articles; 1 set of minutes	14 sets of minutes; 20 articles	19 sets of minutes + 23 texts = 41

* The total number of references to dementia praecox does not correspond to the total number of texts, since one single text may simultaneously cite both of these diagnostic categories.

Source: Survey conducted by Ana Teresa A. Venancio.

One of the characters in these discussions was Murillo de Souza Campos. Campos adopted and defended the diagnosis of schizophrenia, basing it on both the psychodynamic psychiatry of Bleuler, analyzed in Venancio (2010), as well as the constitutionalist theory of Kretschmer, examined by Beraldo (2021). Murillo de Campos received his degree in medicine from the Rio de Janeiro Medical School in 1908, defended his doctoral thesis in medicine entitled *Dores torácicas* [Thoracic Pain], and the following year became a physician with the Health Service at the Central Army Hospital (1909-1932). According to Beraldo (2021), by the end of the 1920s Murillo de Campos had a successful career as an army doctor. He wrote in the *Boletim da Sociedade Médico-Cirurgica Militar* (1915-1920) and the *Revista de Medicina e Higiene Militar* (1921-1931), worked in military scientific institutions and participated in the Rondon Commission (1910-1912), and maintained his interest in psychiatry alongside his military career.

During the 1920s, Murillo de Campos’s career led him to write about the medical service within the army, which also described his work in the institutionalization and modernization of this service, encouraging specialization among military physicians (Campos, 1925; Campos, Moraes, 1923; Cardoso, 2013, p.352-358; Beraldo, 2021, p.255-257). During this period he also became interested in “rural diseases” like beriberi, presenting in congresses and publishing with Juliano Moreira (Campos, Carvalho, Moreira, 1916, p.306), who had examined this topic in the 1890s (Moreira, 1898). Campos addressed other mental health topics besides schizophrenia in his texts: mental health problems in the armed forces, delirium, alcoholism, affectivity, and paranoia (in 1913, 1924, 1926, 1931, and 1935, respectively). Throughout this period, he also participated in SBPML sessions, discussing works presented there by his colleagues on all these topics. From 1930, Murillo de Campos went on to write several texts on spiritism, considering its psychological, clinical, and medical/legal aspects.

In the area of associations, in the late 1910s he became a member of the SBPNML (1917), the Brazilian Mental Hygiene League (1923), and the Brazilian Psychoanalysis Society (1929). “Murillo de Campos was part of the circle of psychiatric physicians who, in the early 1920s, disseminated psychoanalysis, proposing it as therapeutic practice and publishing on it and publicizing it along the way” (Venancio, 2010, p.332). His military career appears to have been truly favorable to position him within clinical psychiatry, since in 1924 he was named head of the psychiatric clinic at the Central Army Hospital. The following year, he became head of the Military Section (Nina Rodrigues Section) of the Hospital Nacional de Alienados, and in 1926 he served in the psychiatric clinic at the Rio de Janeiro Medical School under the command of the Ministry of War.

In 1928, Campos defended his dissertation for consideration as a professor at the medical school, entitled *As constituições em psiquiatria: contribuição ao seu estudo* [The Constitutions in Psychiatry: Contributions to Their Study], and became a lecturer in clinical psychiatry at this institution in 1929. The use of schizophrenia as a category at the Observation Pavilion in the Hospital Nacional de Alienados expanded at the same time that Murillo de Campos entered this institution (1926). During this time (1926-1928), Murillo de Campos, who was then assistant to Henrique Roxo, was conducting clinical observations for his teaching thesis, using the concept of schizophrenia (Beraldo, 2021, p.257). As he stated in his dissertation, Murillo de Campos was firmly rooted in the work of Ernst Kretschmer from the fifth and sixth editions (1926) of his 1921 text *Körperbau und Charakter*, reproducing the methodological system (“Constitution Record Sheet”) used to investigate the relationships between body structures (face and skull, torso and limbs, body surface, glands, and viscera) and psychological disposition. “By the diagrammed description, by measurements and by photography, the diagnostic of body structure is attained, a fundamental somatic [trait] in the psychiatric constitution” (Campos, 1928, p.25). In this constitutional system, body and mind comprised an individual totality.

For his dissertation, Murillo de Campos examined 83 patients (aged 17 to 64), divided between 43 schizophrenics (23 males, 20 females) and 40 manic-depressives (20 males, 20 females). Briefly, Campos recognized that his group was not a homogeneous one like that studied by Kretschmer in his first observations in Swabia (a region in Germany). Still, he argued that the observations present in his work generally did not stay far from the facts established by Kretschmer, “that there is a biological affinity between the pyknic body structure and the psychological disposition for manic-depressive psychosis, on the one hand, and between asthenic/athletic/dysplastic structure and schizophrenia, on the other” (Campos, 1928, p.105-106).

Unlike Almeida (1917, 1920) and Austregésilo (1918), Murillo de Campos utilized the category of schizophrenia at various points in his dissertation. He appropriated Bleuler’s concept by incorporating Kretschmer’s theoretical contributions. The recurring presence of categories such as schizophrenia and schizoid temperament in Murillo de Campos’s dissertation are signs that reinforce the interconnected relationships between Bleuler’s psychodynamics and Kretschmer’s constitutionalism, to delineate mental illness in the late 1920s within the psychiatric context of Rio de Janeiro.

## Final considerations

This article contributes to the understanding of how psychiatric diagnoses and categories historically emerge, are defined, and circulate, focusing on the specific case of schizophrenia within the psychiatric context of Rio de Janeiro during the early twentieth century. We observed initial critiques of schizophrenia as a category in the face of the hegemonic diagnosis of dementia praecox, expressed by various psychiatrists in the scientific literature as well as in care practices. We noted that in the late 1920s the diagnosis of schizophrenia circulated significantly in important scientific and care institutions in Rio de Janeiro to the detriment of dementia praecox, through presentations at congresses, use of the new diagnosis in medical records, clinical discussions in scientific societies, specialized articles, and dissertations. In other words, both the Observation Pavilion at the HNA and SBPNML, through its meetings, were part of the movement to appropriate the category of schizophrenia from where it was circulating in French, German, and Spanish institutional spaces.

We also demonstrated that the incorporation of schizophrenia as a category in these spaces took advantage of the dialog in the European context between Bleuler’s ideas about psychodynamics and Kretschmer’s constitutionalist theory, with references to these authors and others who cross-referenced both, which circulated within Brazil through reading and citation of the originals as well as translation and appropriation in French and Spanish. We consequently underscore the importance of this plural linguistic circuit expressed in the episodes of circulation and appropriation of schizophrenia and related terminology in Brazilian psychiatry in the early twentieth century. Similar to what occurred in French, German, and Spanish institutional spaces, Brazilian psychiatrists actively participated in the transnational circulation of the category of schizophrenia by appropriating it and more broadly using related language in accordance with local interests.

## NOTES

1 Here we refer to both classic texts in the history of psychiatry that construct a *whig* history (Alexander, Selesnick, 1980), related to the critical history of the clinic (Bercherie, 1980; Noll, 2011), as well as analyses by Ibero-American authors on the diagnosis of schizophrenia (Novella, Huertas, 2010; Conti, 2010; Álvarez, Colina, 2011; Golcman, 2015).

2
*Arquivos Brasileiros de Neuriatria e Psiquiatria* [Brazilian Archives of Neuriatry and Psychiatry] was initially published in 1905 under the name *Arquivos Brasileiros de Psiquiatria, Neurologia e Ciências Afins* [Brazilian Archives of Psychiatry, Neurology, and Related Sciences]. In 1907 it was renamed *Arquivos Brasileiros de Psiquiatria, Neurologia e Medicina Legal* [Brazilian Archives of Psychiatry, Neurology, and Legal Medicine], and was linked to the scientific society of the same name which was inaugurated that year. In 1919 the publication’s name was changed again, this time to *Arquivos Brasileiros de Neuriatria e Psiquiatria* [Brazilian Archives of Neuriatry and Psychiatry].

3 The National Asylum for the Insane (Hospício Nacional de Alienados) was the first care institution specialized in psychiatry in Brazil; it was founded in 1852 during the Imperial period as the Pedro II Asylum (Hospício de Pedro II), and renovated at the start of the Republican era, when it was renamed. In 1911, it began to be called the National Hospital for the Insane (Hospital Nacional de Alienados). It is the psychiatric institution which has been most thoroughly researched by the national historiography; one of the most recent publications on this subject is a compilation by Venancio & Dias (2022).

4 In this and other citations of texts from non-English languages, a free translation has been provided.

5 In Brazil, Henrique Roxo, Murillo de Campos, Heitor Carpinteiro Péres, and Augusto Luís Nobre de Mello, members of the SBPNML, read and discussed Minkowski’s work.

6 On the history of this pavilion and its relationships with the National Asylum for the Insane, see Venancio (2003) and Mathias (2017, 20, 2022).

8 Note that the number of entries does not exactly express the number of patients admitted to the HNA, since patients who passed through the Observation Pavilion may not have been referred for prolonged admission to the HNA. Furthermore, during the period of study the same patient may have been admitted more than once to the Observation Pavilion.
