# Supplementary material for: Circulation of ideas about schizophrenia within the psychiatric context of Rio de Janeiro in the 1910s and 1920s
Source: Hist Cienc Saude Manguinhos. 2024 Nov 4;31:e2024057. doi: 10.1590/S0104-59702024000100057en (PMC11534147; doi:10.1590/S0104-59702024000100057en)
Supplement: Translation

# Translation: A circulação de ideias sobre a esquizofrenia no contexto psiquiátrico do Rio de Janeiro, décadas de 1910 e 1920

**DOI:** 10.1590/S0104-59702024000100057

**Authors:** Ana Teresa A. Venancio, Renilson Beraldo

**Affiliations:** i Pesquisadora, Programa de Pós-graduação em História das Ciências e da Saúde/Casa de Oswaldo Cruz/Fiocruz. Rio de Janeiro – RJ – Brasil. ana-teresa.venancio@fiocruz.br; ii Doutor em História das Ciências e da Saúde, Casa de Oswaldo Cruz/Fiocruz. Rio de Janeiro – RJ – Brasil. beraldo_rfs@hotmail.com

**Keywords:** Esquizofrenia, Paul Eugen Bleuler (1857-1939, Ernst Kretschmer (1888-1964, Rio de Janeiro, Circulação de ideias

## Abstract

O artigo examina a circulação da categoria esquizofrenia em debates e espaços psiquiátricos no Rio de Janeiro no início do século XX. Analisa essa categoria no intercâmbio científico entre Eugen Bleuler e Ernst Kretschmer – observando correspondências entre teorias psicodinâmicas e constitucionalistas na Europa – para então perseguir seu uso no Pavilhão de Observação do Hospital Nacional de Alienados, em artigos e reuniões científicas. Investigamos como sua circulação foi simultânea nos espaços científicos e assistenciais e também demonstramos que, como ocorrera no contexto europeu, os psiquiatras brasileiros participaram ativamente da circulação transnacional da categoria esquizofrenia por meio de sua apropriação e pelo uso ampliado do campo semântico “esquizo” segundo interesses locais.

Desde sua criação como especialidade médica, na aurora do século XIX, a psiquiatria tem se dedicado ao problema da relação entre corpo e mente na definição das patologias mentais. Seus desenvolvimentos subsequentes levaram à formulação de um conjunto de discursos e teorias que, em cada período e lugar histórico, tem buscado definir o que deve ser visto como fenômeno relativo à patologia mental: uma patologia ao mesmo tempo físico-moral, que se distinguiria daquelas especificamente orgânicas, mas que não se cansa de buscar a comprovação de seu substrato corpóreo. Devido a essa natureza específica da patologia que visa tratar, a psiquiatria não é uma especialidade médica como as outras. A tensão ou oscilação entre ideias, teorias ou terapêuticas psiquiátricas mais preocupadas com a dimensão física do ser humano, e outras que privilegiam a análise da dimensão moral ou psicológica (essa outra dimensão contrastiva à corpórea), tem sido uma constante na história da psiquiatria, tanto diacrônica quanto sincronicamente.

Partindo dessa perspectiva sobre o objeto psiquiátrico, vamos apresentar o modo como a categoria esquizofrenia – cunhada pelo psiquiatra suíço Paul Eugen Bleuler (1857-1939) (Bleuler, 1911) – circulou no contexto do Rio de Janeiro (Brasil), traduzindo ideias relativas à possibilidade cultural moderna de pensar a existência de um sujeito cindido em si mesmo. Acompanhamos aqui a historiografia internacional^1^ que afirma a importância da relação entre psiquiatria e psicanálise para a criação do referido diagnóstico, fundado na ideia de um psiquismo dinâmico; mas também destacamos a contribuição de teorias relativas à corporalidade em sua dimensão anátomo-orgânica para a afirmação e circulação da esquizofrenia na psiquiatria brasileira, a exemplo do que ocorreu em outros países. Assim, por meio dos debates em torno da esquizofrenia, observamos a divulgação e circulação de ideias advindas da psicanálise e de sua ênfase na dimensão psíquica dos indivíduos, associadas a teorias constitucionalistas do psiquiatra Ernst Kretschmer (1888-1964).

Em nossa perspectiva, essa aparente dualidade de perspectivas expressa uma correlação entre teorias psicodinâmicas e constitucionalistas exatamente porque estas últimas favoreciam abordar a relação entre corpo e mente. Partindo de uma ideia de totalidade individual, apoiadas no holismo médico e nas concepções de unidade orgânica e de unidade físico-moral, as teorias constitucionalistas de Kretschmer incluíam um olhar e interesse sobre a própria dimensão psíquica necessária ao debate das chamadas patologias mentais. Nesse sentido, observamos que a dimensão moral psicológica da nova categoria – esquizofrenia – com sua ênfase no dinamismo da psique foi, ao mesmo tempo, lida e difundida a partir de uma visão holista sobre o organismo.

Nosso objetivo, portanto, é apresentar como o diagnóstico de esquizofrenia, cunhado por Bleuler e expressão das teorias psicodinâmicas, circulou e foi apropriado no campo psiquiátrico na cidade do Rio de Janeiro por meio dos debates científicos e práticas assistenciais de duas de suas importantes instituições na época: a Sociedade Brasileira de Psiquiatria, Neurologia e Medicina Legal, por intermédio de seu periódico científico *Arquivos Brasileiros de Neuriatria e Psiquiatria*,^2^ primeira publicação brasileira especializada na matéria psiquiátrica, e o Pavilhão de Observação do Hospício Nacional de Alienados,^3^ que, desde o início da década de 1890, serviu, ao mesmo tempo, como porta de entrada da assistência e como lugar de ensino e pesquisa da clínica psiquiátrica da Faculdade de Medicina do Rio de Janeiro (FMRJ).

Neste artigo, compartilhamos do pressuposto de Secord (2004, p.661-662) de que o conhecimento pode ser observado na forma de ações comunicativas. Ou seja, em nosso caso, os debates científicos (reuniões, conferências, artigos) e as práticas assistenciais são compreendidos como atos de comunicação da ciência. Por sua vez, segundo Raj (2013, p.344), tais atos contribuem para a constituição de espaços de circulação. Dessa forma, em diálogo com esses autores, entendemos que a circulação de conhecimento decorre do contato entre pessoas, instituições e ideias. Tal contato não apenas auxiliou na estruturação de categorias diagnósticas, mas, especificamente a partir do exame do diálogo Bleuler-Kretschmer, essa estruturação sobrepujou fronteiras nacionais e linguísticas informando uma circulação transnacional de conhecimentos (Weinstein, 2013, p.17). Assim, não miramos o papel de atores isolados como pontos de origem ou potenciais consumidores de teorias e conceitos. Outrossim, visualizamos os contextos/espaços de circulação e apropriação do campo semântico “esquizo” condizentes a interesses e configurações intelectuais específicas (Patiniotis, 2013, p.773-774).

## Diálogos entre Bleuler e Kretschmer na circulação da categoria esquizofrenia

Ao criar a categoria esquizofrenia, o psiquiatra suíço Eugen Bleuler era diretor do Sanatório de Burghölzli (hospital universitário em Zurique), onde trabalhava mais estreitamente com Carl Gustav Jung (1875-1961), que, jovem na época, apresentara os trabalhos de Sigmund Freud a Bleuler. É nesse contexto de encontro entre psicanálise e psiquiatria, no início do século XX, em que se constituiu uma “psiquiatria psicodinâmica”, que a nova categoria emerge. O intuito de Bleuler era substituir o diagnóstico de demência precoce, criado pelo psiquiatra alemão Émil Kraepelin (1856-1925) no fim do século XIX e amplamente utilizado nos hemisférios Sul e Norte. Segundo a historiografia citada, o surgimento da categoria esquizofrenia ocorre em 1911, quando Bleuler publica seu livro seminal *Dementia praecox or The group of schizophrenias.* É definida por ele com base em quatro sintomas fundamentais: distúrbios do pensamento, dissociação ideoafetiva, ambivalência e autismo – este último termo tendo sido cunhado pelo próprio Bleuler para falar de fuga da realidade. A partir de então, o psiquiatra suíço faz circular a nova categoria em suas publicações, como em *Lehrbuch der Psychiatrie* [Tratado de psiquiatria, de 1917], e apresentações em congressos, como “La schizophrénie”, no Congrès des Médecins Alienistes et Neurologistes de France et des Pays de Langue Française, em Genebra e Lausanne, em 1926, onde o francês Henri Claude (1869-1945) também apresentava trabalho sobre o tema. Bleuler aderia a alguns princípios básicos da psiquiatria psicodinâmica, como a ideia de um psiquismo em movimento, atuante, também por sua dimensão inconsciente, ainda que tenha mantido reserva quanto à ideia freudiana da centralidade da sexualidade e sua significação nos processos psíquicos.

Dez anos depois do livro de Bleuler, Kretschmer publicou a primeira edição de uma de suas obras mais traduzidas, *Estrutura corporal e caráter* [*Körperbau und Charakter*, 1921]. Partindo de duas patologias mentais clássicas criadas por Kraepelin – demência precoce e psicose maníaco-depressiva –, Kretschmer correlacionou elementos corporais (anatômicos, fisiológicos, metabólicos e endocrinológicos) com características de temperamento (ciclotimia e esquizotimia; cicloidia e esquizoidia), estabelecendo uma dupla tipologia constitucional em que as características temperamentais e morfológicas de indivíduos maníaco-depressivos e esquizofrênicos poderiam ser encontradas em indivíduos sãos num estágio “atenuado” (Bercherie, 1980, p.159-168). Com isso, Kretschmer estabelecia duas operações analíticas importantes: fornecia instrumental teórico para a análise da correlação físico-moral e relativizava as fronteiras entre fenômenos psicopatológicos e estados mentais considerados saudáveis.

Embora tenha baseado suas pesquisas iniciais em categorias kraepelinianas, Kretschmer foi bastante criticado pelos integrantes do Instituto Alemão de Pesquisa em Psiquiatria, de Munique [Deutschen Forschungsanstalt für Psychiatrie], incluindo o próprio Kraepelin (Engstrom, 2008, p.241). O acusavam de ser muito “psicológico” e de não se apropriar do “biológico” em suas observações, além de defender o fim da separação estrita entre causas endógenas e psicogênicas (Priwitzer, 2007, p.281-282).

No caso da esquizofrenia, o diálogo de Kretschmer será mais próximo a Bleurer, que não escondia sua admiração por Kretschmer desde quando este fora discípulo de Robert Gaupp (1870-1953) em Tubinga em meados de 1918 (Beraldo, 2021, p.179). Segundo Patrício (2001, p.86-88), Kretschmer aprendeu com Gaupp a abordagem caracterológica em que a manifestação prévia de uma enfermidade poderia ser buscada em traços de personalidade do indivíduo. A adesão a essa abordagem foi importante, pois favoreceu suas descrições das personalidades anormais esquizoides e cicloides correlacionadas respectivamente à esquizofrenia e à psicose maníaco-depressiva, como formas atenuadas destas últimas.

Para Kretschmer, uma abordagem diagnóstica deveria abarcar a dimensão psicofísica do indivíduo em sua totalidade, a qual seria formada por três aspectos. O primeiro deles seria a “constituição”, que expressava a confluência entre o somático e o psíquico. Nesse sentido, os tipos constitucionais por Kretschmer falam da relação entre corpo, temperamento e caráter: o “temperamento” estava relacionado à fisiologia nervosa e endócrina; enquanto o “caráter” dizia respeito à dimensão psicológica do indivíduo, formada por experiências e vivências prévias ao surgimento da psicose. As experiências e vivências, assim, poderiam operar como “situações-chave”, responsáveis pelo aparecimento da patologia. Para Kretschmer, portanto, a estrutura, a figura ou o hábito corporal (propriedades do organismo) seriam expressões/reações da constituição individual que possuía uma base hereditária (Beraldo, 2021, p.177-179). Nessa perspectiva, a agenda de pesquisas de Kretschmer procurava determinar a frequência com que certas formas psiquiátricas (esquizofrenia e psicose maníaco-depressiva) poderiam ser correlacionadas a certos tipos de estrutura corporal.

Kretschmer (1923) também continuaria discutindo a respeito da sede ou residência do espírito ou alma, incrementando suas reflexões publicadas desde 1920 em sua obra *Psicologia médica* [*Medizinische Psychologie*] (Kretschmer, 1957). Fundamentando-se em autores neovitalistas, como o filósofo e biólogo Hans Driesch (1867-1941), e em contribuições da psiquiatria, como Bleuler, considerava que a psiquê não estaria ligada a funções exclusivas de um ou outro órgão – como o cérebro ou tronco encefálico –, mas teria um substrato tanto anatômico como fisiológico, considerando o corpo em sua integralidade (Kretschmer, 1957, p.11-12). Essa visão integral antecede o livro mais conhecido de Kretschmer (1921). Seu interesse pela dimensão moral e psicológica também se expressava pelo uso de noções como traços de caráter e tipos de reação, a partir do desenvolvimento da técnica de diagnóstico multidimensional [*Mehrdimensionalen Diagnostik*] (Engstrom, 2008; Priwitzer, 2007).

A partir da historiografia e de fontes primárias, podemos observar que Bleuler e Kretschmer mantiveram trocas intelectuais e se autorreferenciavam desde o pós-Primeira Guerra Mundial. Kretschmer iniciou suas publicações por volta de 1914 em temas como delírios e histeria (1914-1917), passando por análises do conceito de inconsciente (1919), no qual dialoga com Bleuler, culminando em sua dissertação de 1918 sobre paranoia e teoria do caráter, bem como seu livro de 1921 sobre constituição (Priwitzer, 2007, p.289-292). O psiquiatra suíço, por sua vez, lera com entusiasmo a tese de habilitação em psiquiatria de Kretschmer (1918) – *Delírio sensitivo de referência* [*Der sensitive Beziehungswahn*] – na qual este examinava a correlação entre temperamento prévio, experiência e situações de vida na determinação de psicoses. O encontro entre ambos, contudo, ocorreu apenas em 1920, durante o 59° Encontro da Associação Suíça de Psiquiatria (Priwitzer, 2007, p.75-76).

Bleuler (1921) foi também um dos primeiros leitores especialistas a resenhar a obra *Estrutura corporal e caráter*, no mesmo ano de sua publicação. Em 1922, no prefácio à segunda edição de sua própria obra, Kretschmer afirmava que os fatos que observara em 1921 já eram parcialmente conhecidos por Bleuler há muito tempo (Kretschmer, 1925, p.13). Kretschmer, na realidade, reforçava o que R. Gaupp havia escrito, em 1921, no prefácio à primeira edição de sua obra, ou seja, que teria sido por intermédio de Bleuler que se tornou viável à psiquiatria a “compreensão mais profunda dos sintomas e dos conteúdos individuais (caráter e história da personalidade pré-psicótica) nos processos esquizofrênicos” (Kretschmer, 1925, p.10). Segundo o próprio Kretschmer, o caminho para o exame psicopatológico de estados fronteiriços entre psicopatia e normalidade, expresso nos esquizoides, teria sido aberto primeiramente por Bleuler (Kretschmer, 1925, p.122).

Outros episódios do intercâmbio entre a escola de Tubinga e a de Zurique aparecem na terceira (1920) e na quarta edições (1923) do *Tratado de psiquiatria* de Bleuler. No primeiro caso, Bleuler (1920, p.403) utiliza a dissertação de Kretschmer de 1918 para falar de “reações primitivas” [*Primitivreaktionen*] como traços de caráter exagerados (gritos, birras, estupor, depressão). No segundo caso, Bleuler amplia o item chamado “A classificação das doenças mentais”, incluindo algumas elaborações de Kretschmer presentes em seu livro de 1921. Seguindo Kretschmer, destaca o papel das constituições e das disposições de caráter (manias de perseguição e grandeza, por exemplo) como predisposições a delírios e doenças, elaborando, por fim, um comentário extenso sobre o diagnóstico multidimensional de Kretschmer e sua contribuição para o estudo dos temperamentos (Bleuler, 1923, p.132-134). Kretschmer, por seu turno, tomou de empréstimo de Bleuler os termos autismo – caracterizado como “o viver dentro de si mesmo” (Kretschmer, 1925, p.147) – e ambivalência, ou seja, “o salto para frente e para trás da emoção e da vontade” (p.177). Tais termos foram mobilizados por Kretschmer para caracterizar o temperamento esquizoide em correlação com as estruturas corporais astênica e atlética.

A circulação de ideias psiquiátricas entre Bleuler e Kretschmer aqui comentadas, em citações bibliográficas entre eles e encontro em congresso, também se fez por meio de apropriações cruzadas dos dois autores presentes em trabalhos de outros psiquiatras bastante citados no contexto brasileiro, como o psiquiatra francês de origem polonesa Eugène Minkowski (1885-1972) e sua esposa, também psiquiatra, Françoise Minkowska-Brokman (1882-1950).^4^ Ambos foram leitores de Kretschmer e assistentes de Bleuler no Burghölzli, a clínica da universidade. Após a Primeira Guerra Mundial, estabelecidos em Paris, Minkowska desenvolveu pesquisas nas quais reconstituiu árvores genealógicas de famílias de lavradores diagnosticados como epiléticos e esquizofrênicos. Com informações obtidas por meio de parentes vivos, descreveu a constituição epileptoide ou gliscroide (Yahn, 1952, p.93-94).

Minkowski, de seu lado, retomou seus estudos médicos, passando também a trabalhar no Hospital St. Anne, em Paris. Em 1925, publicou “La genèse de la notion de schizophrénie et ses caracteres essentiels” na revista *Evolution Psychiatrie*, e, no ano seguinte, defendia sua tese de doutorado em medicina, *La notion de perte de contact avec la réalité et ses applications en psychopathologie*, fortemente influenciado pelas teorias de Henri Bergson e sob supervisão de Henri Claude, psiquiatra cujas ideias também estiveram presentes na circulação e apropriação sobre a esquizofrenia no contexto científico especializado brasileiro. Os estudos de Minkowski defendiam que as investigações psiquiátricas de caráter constitucional deveriam se orientar para o passado do enfermo e para encontrar nas particularidades preexistentes de caráter (leia-se a dimensão psicológica do indivíduo, formada por experiências e vivências prévias à psicopatologia) os traços essenciais da psicose atual, tal como observavam Bleuler e Kretschmer. Tais aportes aparecem, por exemplo, na obra *La schizophrénie* (Minkowski, 1927). Conforme Silva Neto (2004, p.51-54), com relação à ideia bleuleriana de esquizofrenia, Minkowski teria destacado a noção “perda de contato vital com a realidade”, dando importância a conceitos como duração e tempo, para discutir as nuanças da experiência esquizofrênica. Para Minkowski, a referida noção se tornava o ponto de junção entre a escola de Zurique (Bleuler) e as ideias de Bergson (Minkowski, 2019, p.59; publicado originalmente em 1926). O que verificamos é que o registro dessa circulação transnacional de ideias e pessoas, bem como a reformulação de conceitos, sinaliza uma visada que se situa bem longe das apropriações das ideias de Kretschmer na década de 1930, em que os tipos constitucionais passaram a ser lidos como indícios da estrutura corporal que revelariam traços indeléveis relativos ao temperamento e caráter do indivíduo numa visada eugênica.

## A circulação da categoria esquizofrenia na psiquiatria carioca na década de 1920

A circulação da categoria esquizofrenia no contexto psiquiátrico brasileiro foi mais significativa a partir da segunda metade da década de 1920; como vamos demonstrar aqui de modo original, ocorreu por meio tanto do conhecimento difundido no principal manicômio do país quanto de debates científicos, expressos em teses acadêmicas, artigos em periódicos especializados, congressos e atas de sociedade científica dedicada à matéria psiquiátrica e à jovem área da neurologia. Para tanto, partimos da historiografia que observa que a apropriação da referida categoria se fez primeiramente via a psiquiatria, embora tenhamos encontrado o uso posterior do campo semântico “esquizo” em trabalhos relativos à neurologia.

Com relação à neurologia, é notável que, em 1918, o neurologista Antônio Austregésilo (1876-1960) faça referência apenas à demência precoce (e não à esquizofrenia) quando escreve artigo na revista médica brasileira *A Patologia Geral: Revista de Medicina e Ciências Afins*, ao discutir o diagnóstico de catafrenia, por ele criado. Esse seu artigo seria publicado no ano seguinte em francês na *Revue Neurologique* e apresentado anos depois, em março de 1926, em sessão da Sociedade de Psiquiatria de Paris, da qual era associado. Em 1918, portanto, Austregésilo estava mais afeito às discussões em torno da categoria demência precoce e sua determinação à deterioração intelectual. Apesar de seu novo diagnóstico – catafrenia – estar centrado no destaque aos estados fronteiriços entre eventos psicopatológicos e estados saudáveis, é somente em sua apresentação de 1926 que se valerá do campo semântico “esquizo”, referindo-se a noções como esquizotimia e esquizoide, que, a exemplo de Kretschmer, reforçavam a relativização entre estados são e patológico (Beraldo, 2021, p.229-233).

A produção da categoria catafrenia, ainda que em consonância com as teorias constitucionalistas de Kretschmer, foi desenvolvida a partir de interesses científicos do contexto local do Rio de Janeiro. De um lado, atendia à necessidade do primeiro catedrático de neurologia da Faculdade de Medicina do Rio de Janeiro de se alinhar às discussões da clínica e da patologia geral dessa instituição a respeito do constitucionalismo, fortalecendo uma visada médica holista, defendida por alguns renomados professores que consideravam deletéria a especialização e a fragmentação crescente da medicina na época (Beraldo, 2021, p.95-139). Não parece casual, portanto, que o primeiro texto de Austregésilo (١٩١٨) fosse publicado na revista médica *Patologia Geral*. De outro lado, a criação original da categoria catafrenia também servia para dar destaque e proeminência à jovem especialidade neurológica, *vis-à-vis* o conhecimento psiquiátrico vigente no Rio de Janeiro. Ainda que no ambiente científico carioca tais especialidades médicas se mantivessem reunidas na Sociedade Brasileira de Psiquiatria, Neurologia e Medicina Legal, também é notável, naquele momento, a existência de concorrências entre elas e a necessidade de espaços em separado para suas discussões, a exemplo das comissões diferenciadas criadas para o trato específico dessas matérias (Cerqueira, ٢٠١٤). Por essas vias, no contexto local, as ciências relativas ao mental se especializavam e paulatinamente se diferenciavam. Assim, o uso do campo semântico “esquizo” pelo catedrático em neurologia só se daria quando a categoria esquizofrenia já circulava mais amplamente no meio especializado psiquiátrico carioca, onde fora primeiramente “recepcionado”.

Com relação ao pioneirismo da psiquiatria na circulação da categoria esquizofrenia em nosso país, Murillo de Souza Campos (1887-1968) confirmava, na época, que Hermelino Lopes Rodrigues (1899-1971) teria sido o autor brasileiro do primeiro trabalho sobre esquizofrenia aqui produzido (Campos, 1929, p.178) que positivava seu uso no campo psiquiátrico. Silveira (2009) também afirma que a introdução da ideia de esquizofrenia se deve ao psiquiatra Lopes Rodrigues, em 1926, com a defesa das teses “Etiopatogenia da demência precoce” e “Estudo clínico das esquizofrenias”, no concurso para professor catedrático de Psiquiatria na Faculdade de Ciências Médicas da Universidade Federal de Minas Gerais, em que foi aprovado. Segundo Silveira (2009), Lopes Rodrigues iniciou seu curso de medicina na Faculdade de Medicina da Bahia, em 1916, e, quatro anos depois, obteve transferência para o Rio de Janeiro, onde terminaria seus estudos, sendo, ao mesmo tempo, aluno de Henrique Britto de Belford Roxo (1877-1969) e discípulo de Juliano Moreira (1873-1933), por ter trabalhado com ele no Hospital Nacional de Alienados. Nesse contexto psiquiátrico, Rodrigues teria tomado conhecimento e se impressionado com os “aspectos psicológicos” contemplados no diagnóstico proposto por Bleuler (Silveira, 2009, p.589).

Venancio (2010), por sua vez, demonstra a existência, em 1929, de um debate sobre os usos das categorias demência precoce e esquizofrenia entre o já consagrado Henrique Roxo – professor catedrático da FMRJ e diretor do Pavilhão de Observação do então Hospital Nacional de Alienados – e Murillo de Campos. Os dois psiquiatras divulgavam seus trabalhos no terceiro Congresso Brasileiro de Neurologia, Psiquiatria e Medicina Legal, realizado no Rio de Janeiro, publicando-os, no mesmo ano, no *Arquivos Brasileiros de Neuriatria e Psiquiatria* (Roxo, 1929; Campos, 1929). Em sua análise, Venancio demonstra como esse processo de designação e definição científica dos diagnósticos de demência precoce e de esquizofrenia se articulava com a produção da diferença entre o que deveria e o que não deveria ser visto como fenômeno relacionado à patologia mental. Por um lado, Henrique Roxo aderia a uma visão estática da doença, privilegiando o conceito kraepeliniano de demência precoce em que as características físico-orgânicas eram mais decisivas para a qualificação da patologia, o que levava a uma deterioração completa do sujeito. Por outro lado, Murillo de Campos enfatizou a característica psicodinâmica da morbidade esquizofrênica, discutindo os aspectos psíquicos que, em sua compreensão, seriam mais relevantes na definição dessa patologia mental (Venancio, 2010, p.341).

Nenhum dos autores citados, no entanto, investiu na análise do conjunto de diferentes atores, processos e lugares de ciência e assistência nos quais o diagnóstico de esquizofrenia circulou nem no modo como tal evento foi informado, ao mesmo tempo, pelas teorias de Bleuler e Kretschmer. Assim, procuramos aqui demonstrar o modo processual pelo qual, a partir de meados dos anos 1920, cresceu o interesse e a presença dessa categoria nos dois principais espaços de institucionalização da psiquiatria do Rio de Janeiro, na época: o Pavilhão de Observação do Hospício Nacional de Alienados e a Sociedade Brasileira de Psiquiatria, Neurologia e Medicina Legal (SBPNML) com o então *Arquivos Brasileiros de Neuriatria e Psiquiatria*, periódico brasileiro especializado na matéria psiquiátrica que divulgava não apenas artigos científicos, mas também as atas da referida Sociedade e as discussões dos casos clínicos apresentados nas reuniões dessa agremiação.

O Pavilhão de Observação, a par de ser o local de triagem para a assistência psiquiátrica – cujo regulamento previa a estada do paciente por um período de até 15 dias –, era também, como já mencionado, lugar onde os alunos interessados na matéria faziam sua formação especifica, assistindo a aulas e apresentações de casos clínicos.^5^ O mapeamento quantitativo^6^ dos diagnósticos concedidos a homens e mulheres internados no Pavilhão de Observação do Hospital Nacional de Alienados entre 1903 e 1930 demonstra um número absoluto de homens (23.451) maior que o de mulheres (15.019), totalizando 38.470 entradas^7^ no Pavilhão, com uma distribuição de entrada de pacientes masculinos nos referidos anos que é semelhante à distribuição de entrada de mulheres. Nesse total de entradas, é possível observar também o uso do diagnóstico de “esquizofrenia” a partir de 1926, tanto entre homens como entre mulheres, ao mesmo tempo que diminui o número de pacientes considerados portadores de “demência precoce”.

**Figura 1:** : Gráfico de diagnósticos de demência precoce e esquizofrenia em relação ao número total de entradas no Pavilhão de Observação do Hospital Nacional de Alienados, homens, 1903-1930 (Fonte: Livros-índices do Pavilhão de Observação do HNA no acervo da biblioteca do Instituto de Psiquiatria da Universidade Federal do Rio de Janeiro)

**Figura 2:** : Gráfico de diagnósticos de demência precoce e esquizofrenia em relação ao número total de entradas no Pavilhão de Observação do Hospital Nacional de Alienados, mulheres, 1903-1930 (Fonte: Livros-índices do Pavilhão de Observação do HNA no acervo da biblioteca do Instituto de Psiquiatria da Universidade Federal do Rio de Janeiro)

E como eram apresentados os referidos diagnósticos pela ciência psiquiátrica no Rio de Janeiro? Os artigos científicos e demais textos publicados (algumas resenhas, uma nota bibliográfica e uma notícia sobre instituição científica), assim como as discussões clínicas da SBPNML também divulgadas no periódico *Arquivos Brasileiros de Neuriatria e Psiquiatria*, estiveram de acordo com movimento semelhante ao que se fazia em relação ao uso do diagnóstico de esquizofrenia no Pavilhão de Observação do Hospital Nacional, com o início de aumento de publicações a respeito a partir da década de 1920. Como sabemos, muitos dos médicos psiquiatras que escreviam sobre o assunto eram também médicos no Hospital Nacional e em seu Pavilhão de Observação, utilizando os casos clínicos para suas pesquisas e teses.

Observa-se assim a maior presença da categoria esquizofrenia a partir dos anos 1920, se comparada ao uso da categoria demência precoce, sem que, entretanto, desapareça por completo a referência a esta última. Logo em 1905, no primeiro ano de publicação de *Arquivos Brasileiros*, é divulgado artigo discutindo a demência precoce. Ao longo das décadas de 1900 e 1910 teremos 23 textos discutindo o diagnóstico de demência precoce, sem referências à categoria esquizofrenia, já que essa só aparecerá no contexto europeu no ano seguinte. Esses textos relacionam a categoria kraepeliniana a diversas patologias – sífilis, tuberculose, demência paranoide e parafrenia –, mas principalmente à loucura maníaco-depressiva, perseguindo-se assim um dos cânones da psiquiatria da época cunhado por Kraepelin, isto é, a distinção entre demência precoce e psicose maníaco-depressiva (Kendler, Engstrom, 2018).

É em artigo de Waldemar de Almeida (1917) que aparece pela primeira vez uma referência a Eugen Bleuler. Almeida formou-se pela FMRJ, em 1909, mas desde o ano anterior foi interno de medicina no Pavilhão de Observação e, na sequência, assistente e médico da Assistência a Alienados até 1922, quando se tornou diretor do Asilo Colônia de Alienados de Vargem Alegre, ao mesmo tempo que manteve consultório particular entre 1915 e 1922 (Cerqueira, 2014, p.30). Em seu texto, ao tratar do diagnóstico, das remissões e dos tratamentos para os dementes precoces, Almeida menciona a controvérsia entre Bleuler e Kraepelin, defendendo, entretanto, as ideias deste último. O texto de Almeida é expressivo do que se observa nas discussões diagnósticas da década de 1910, em que prevaleciam tanto a crítica à amplitude do conceito de Bleuler exemplificada na citação abaixo de Austregésilo (1918) quanto a consolidação do uso do diagnóstico de demência precoce, conforme o próprio resumo que Almeida (1917, p.73) apresenta de seu texto: “Neste trabalho o autor depois de realçar o papel da demência precoce criada por Kraepelin, lembra uma rápida síntese das críticas, os adeptos e os opositores que teve tal entidade, para se firmar definitivamente na patologia mental”. E ainda: “O conceito de Bleuler acerca da esquizofrenia ainda se não acha bem definido, porque, revendo os estudos do psiquiatra de Zurich, noto que houve grande ampliação de limites, baseados mais em estudos da psicanálise do que na clínica propriamente dita” (Austregésilo, 1918, p.124).

A década de 1920, após a Primeira Guerra Mundial, apresentou grande circulação de ideias e atores nos campos da psiquiatria e da neurologia no Brasil, estreitando-se laços com o contexto germânico e retomando-se contatos com médicos alienistas franceses (Venancio, Cerqueira, 2016, p.13-15, 22), como o neurologista Joseph Babinski e o psicólogo George Dumas, este último já mantendo intercâmbios culturais e científicos com psiquiatras brasileiros desde antes da Primeira Guerra Mundial. No caso da Alemanha, o intercâmbio científico que já ocorria antes da guerra, com esforços dos alemães em relação aos países ibero-americanos, foi ampliado após o conflito mundial, com o crescente interesse dos médicos brasileiros em participar de cursos de especialização nos centros universitários e hospitais psiquiátricos alemães, trazendo especialistas para realizar palestras e cursos no Brasil (Venancio, Cerqueira, 2016, p.22, 33; Muñoz, 2018, p.140-141).

Junto a essas atividades científicas, observa-se a circulação de livros e artigos especializados, sendo possível destacar que, além dos franceses, muitos dos autores citados pelos médicos cariocas em matérias de interesse psiquiátrico eram alemães, mas também, como demonstra Beraldo (2023), havia intercâmbios entre psiquiatras espanhóis e brasileiros, bem como referências a psiquiatras do contexto germânico traduzidos por espanhóis. Murillo de Campos, nesse sentido, é um ótimo exemplo desse circuito de ideias em movimento a respeito da esquizofrenia. Ao objetivar acompanhar as ideias de Bleuler, Campos (1929) cita trabalhos do psiquiatra suíço editados em várias línguas: *Dementia precox oder Gruppe der Schizophrenien*, de 1911; *Tratado de psiquiatria*, de 1924; e “La schizophrénie”, apresentado no Congrès des Médecins Alienistes et Neurologistes de France et des Pays de Langue Française, em outubro de 1926. Além disso, no mesmo artigo se utiliza de Kretschmer (no original em alemão) e Eugène Minkowski (em francês), ao mesmo tempo que faz referência a outros autores germânicos traduzidos para o espanhol, como o *Tratado de las enfermedades mentales*, organizado por Oswald Bumke (1877-1950) e publicado em Barcelona em 1926; e a obra de Hanz Walter Gruhle (1880-1958) *La psiquiatria* – coeditada em Barcelona e Buenos Aires em 1925.

Nessa perspectiva, a circulação das teorias de Bleuler e Kretschmer fora do contexto germânico, por meio de versões em outras línguas, potencializou seu uso, diminuindo a distância linguística, elemento importante para o intercâmbio científico, como apontam Burke e Hsia (2009, p.13-17). Com relação especificamente a Kretschmer, Beraldo (2021) aponta que o próprio psiquiatra teria divulgado em espanhol uma síntese de suas ideias na prestigiosa *Revista de Occidente* (1923). Fundada pelo ensaísta e filósofo espanhol José Ortega y Gasset (1883-1955), em Madrid, em 1923, circulou até o início da Guerra Civil Espanhola, reunindo um número seleto e diversificado de colaboradores estrangeiros – o físico Albert Einstein, o filósofo Max Scheller, o sociólogo Georg Simmel, o poeta Pablo Neruda – e espanhóis, como o filósofo Fernando Vela (cofundador da revista), o endocrinologista Gregório Marañon, o psiquiatra José Miguel Sacristán, entre outros. A divulgação da síntese das ideias kretschmerianas, portanto, ocorria num espaço de divulgação de temas científicos variados, além de novidades das áreas de filosofia, literatura, estética e textos relativos à conjuntura de decadência e crise do ambiente europeu no pós-Primeira Guerra Mundial (Linacero, 2000, p.379).

Foi nessa zona de circulação de ideias, autores e debates diagnósticos no contexto psiquiátrico do Rio de Janeiro que, ao longo da década de 1920, se observa o uso mais ampliado da categoria esquizofrenia. Durante os anos 1920, o periódico *Arquivos Brasileiros de Neuriatria e Psiquiatria* publica seis artigos com as primeiras referências à esquizofrenia. Os primeiros escritos – Almeida (1920); Conrado (1921) – não a tratam como uma categoria diagnóstica, mas como “forma” ou “sintoma” possível de existir em casos de sífilis cerebral. O texto de Almeida (1920) “Sífilis cerebral de forma esquizofrênica” era de fato um comentário de um parágrafo sobre texto de mesmo título publicado no *Arquivos Rio-grandenses de Medicina* em janeiro daquele ano. O texto subsequente (Conrado, 1921) também se refere mais a uma forma que a um diagnóstico: “Estudo clínico da forma esquizofrenoide da sífilis cerebral”. Trata-se de um trabalho decorrente das observações clínicas da Seção Pinel do Hospital Nacional de Alienados, que era chefiada por Ulisses Viana (1880-1939). Desde a década de 1910, Vianna, sua equipe e seus parceiros estavam interessados nas pesquisas sobre sífilis, incluindo suas relações com a demência precoce (SBPNML, 1914; Vianna, Moses, 1919). Até os primeiros anos da década de 1920, portanto, as referências à esquizofrenia, quando presentes, têm mais o estatuto de sintomas do que de uma entidade nosológica, por vezes aparecendo, assim como a demência precoce, como tema secundário em relação à discussão central de outros diagnósticos protagonistas de pesquisas na época, como a chamada sífilis cerebral.

Na sequência desses artigos de 1920 e 1921, encontramos no periódico *Arquivos Brasileiros de Neuriatria e Psiquiatria* a publicação de várias atas de sessões ordinárias da SBPNML dos anos de 1920, 1923, 1924 e 1926 a 1929 com menção a estados patológicos relativos ao campo semântico “esquizo”. As atas traduziam, na verdade, o que o relator selecionava das discussões presenciais sobre assuntos diversos, incluindo as discussões de casos clínicos que ocorriam nas reuniões da referida sociedade. Nesse sentido, não podemos dizer que revelavam na íntegra o conteúdo dos debates então ocorridos entre pares, embora certamente fosse significativo que, no caso em estudo, uma quantidade cada vez maior de referências à esquizofrenia estivesse na ordem do dia no campo psiquiátrico carioca, em textos de tipos diversos, incluindo o debate de casos clínicos advindos, em grande parte, do Hospício Nacional de Alienados: “O Dr. Xavier de Oliveira apresenta dois casos, um de esquizofrenia, e outro de demência precoce. Discorre ligeiramente sobre a constituição esquizofrênica e termina perguntando se a ‘demência precoce’ é a mesma coisa que a ‘esquizofrenia’” (SBPNML, 1927, p.178-179; destaques no original).

**Tabela 1:** : Frequência de textos em Arquivos Brasileiros de Neuriatria e Psiquiatria sobre demência precoce e esquizofrenia

^**Período**^	^**Referências principais à demência precoce**^	^**Referências principais à esquizofrenia**^	^Número total de textos e atas*^
1905-1909	4 textos	-	4
1910-1919	7 textos; 12 atas de reuniões da SBPNML	1 artigo (1917)	7 textos +12 atas = 19
1920-1929	3 artigos; 6 atas	6 artigos; 4 atas	7 textos +10 atas = 17
1930-1939	2 artigos; 1 ata	14 atas; 20 artigos	19 atas + 23 textos = 41

* A soma das referências à demência precoce e à esquizofrenia não corresponde ao número total de textos, já que um mesmo texto pode estar citando concomitantemente as duas categorias nosológicas.

Fonte: Levantamento realizado por Ana Teresa A. Venancio.

Um dos personagens atuantes nessas discussões foi Murillo de Souza Campos, que adotou e defendeu o diagnóstico de esquizofrenia, fundamentando-se tanto na psiquiatria psicodinâmica de Bleuler, já analisada em Venancio (2010), quanto na teoria constitucionalista de Kretschmer, pesquisada por Beraldo (2021). Formado em medicina, pela FMRJ, em 1908, defendeu a tese de doutorado em medicina *Dores torácicas*, tornando-se no ano seguinte médico do Serviço de Saúde do Hospital Central do Exército (1909-1932). Segundo Beraldo (2021), até final da década de 1920, Murillo de Campos teve uma carreira profícua como médico do Exército. Publicou no *Boletim da Sociedade Médico-Cirurgica Militar* (1915-1920) e na *Revista de Medicina e Higiene Militar* (1921-1931), atuou em instituições científicas militares e participou da Comissão Rondon (1910-1912), mantendo seu interesse na área psiquiátrica em concomitância com sua carreira militar.

Ao longo da década de 1920, a carreira médico-militar de Murillo de Campos o levou a escrever sobre o próprio serviço médico no Exército, o que também falava a favor de sua atuação na institucionalização e modernização desse serviço, com o estímulo à especialização dos médicos militares (Campos, 1925; Campos, Moraes, 1923; Cardoso, 2013, p.352-358; Beraldo, 2021, p.255-257). Também nesse período se interessou pelas “doenças do interior do país” – como o beribéri –, apresentando trabalho em congresso e publicando a respeito com Juliano Moreira (Campos, Carvalho, Moreira, 1916, p.306), que já havia se debruçado sobre o assunto no final da década de 1890 (Moreira, 1898). No que se refere ao campo da medicina mental, além do tema da esquizofrenia, os textos de Campos (1913, 1924, 1926, 1931, 1935) tratavam dos seguintes assuntos: moléstias mentais nas forças armadas, delírio, alcoolismo, afetividade e paranoia. Ao longo desse período, também participou das sessões da SBPML, discutindo trabalhos ali expostos, de seus colegas, sobre todos esses temas. A partir de 1930, Murillo de Campos vai desenvolver vários trabalhos sobre o espiritismo, considerando seus aspectos psicológicos, clínicos e médico-legais.

No âmbito associativo, a partir no final da década de 1910, tornou-se membro da SBPNML (1917), da Liga Brasileira de Higiene Mental (1923) e da Sociedade Brasileira de Psicanálise (1929). “Murillo de Campos fazia parte do círculo de médicos psiquiatras que, naquele início dos anos de 1920, passavam a difundir a psicanálise, propondo-a como prática terapêutica, publicando trabalhos a respeito dela e difundindo-a em curso” (Venancio, 2010, p.332). A carreira militar parece mesmo ter concorrido favoravelmente para a sua inserção na clínica psiquiátrica, já que em 1924 foi nomeado chefe da Clínica Psiquiátrica do Hospital Central do Exército. No ano seguinte, tornou-se chefe da Sessão Militar (Sessão Nina Rodrigues) do Hospital Nacional de Alienados, e, em 1926, passou a servir na Clínica Psiquiátrica da FMRJ, sob ordem do ministro da Guerra.

Em 1928, Campos defenderá a sua tese de livre-docência na FMRJ – *As constituições em psiquiatria: contribuição ao seu estudo* –, tornando-se docente de clínica psiquiátrica dessa faculdade a partir de 1929. O aumento do uso da categoria esquizofrenia no Pavilhão de Observação do Hospital Nacional de Alienados foi concomitante à inserção de Murillo de Campos naquela instituição (1926). Foi nesse intervalo de tempo (1926-1928) que Murillo de Campos, então assistente de Henrique Roxo, realizou observações clínicas para a sua tese de livre-docência, utilizando o conceito de esquizofrenia (Beraldo, 2021, p.257). Conforme expressa sua tese, Murillo de Campos ancora-se no trabalho de Ernst Kretschmer a partir da quinta e da sexta edições (1926) da sua obra *Körperbau und Charakter*, de 1921, reproduzindo a sistemática metodológica (“Ficha Constitucional”) utilizada para a investigação das relações entre a estrutura corporal (face e crânio, tronco e membros, superfície corporal, glândulas e vísceras) e a disposição psíquica. “Pela descrição diagramática, pelas mensurações e pelas fotografias, chega-se ao diagnóstico da estrutura corporal, fundamento somático da constituição psiquiátrica” (Campos, 1928, p.25). Portanto, nessa sistemática constitucional, corpo e mente compõem uma totalidade individual.

Para a confecção de sua tese, Murillo de Campos examinou 83 enfermos (entre 17 e 64 anos), divididos entre 43 esquizofrênicos (23 do sexo masculino, 20 do sexo feminino) e 40 maníaco-depressivos (20 do sexo masculino, 20 do sexo feminino). Em resumo, Campos reconheceu que seu material não constituía um conjunto homogêneo, tal qual aquele estudado por Kretschmer em suas primeiras observações na Suábia (região da Alemanha). Por outro lado, argumentava que as observações presentes em seu trabalho, no objetivo geral, não se afastavam dos fatos estabelecidos por Kretschmer “de que há uma afinidade biológica entre a estrutura corporal pícnica e a disposição psíquica própria à psicose maníaco-depressiva, por um lado, e entre a estrutura astênico-atlético-displásica e a esquizofrenia, por outro” (Campos, 1928, p.105-106).

Diferentemente de Almeida (1917, 1920) e Austregésilo (1918), Murillo de Campos mobilizou a categoria esquizofrenia em vários momentos de sua tese. Sua apropriação do conceito de Bleuler ocorrera mediante a incorporação dos aportes teóricos de Kretschmer. A presença recorrente de categorias como esquizofrenia e temperamento esquizoide na tese de Murillo de Campos são indícios que reforçam as inter-relações entre a elaboração psicodinâmica bleuleriana e constitucionalista kretschmeriana para a circunscrição das enfermidades mentais no final da década de 1920 no contexto psiquiátrico do Rio de Janeiro.

## Considerações finais

Neste artigo buscamos contribuir para o entendimento do modo como, historicamente, surgem, são definidos e circulam diagnósticos e categorias psiquiátricos, pondo em análise o caso específico da esquizofrenia no contexto psiquiátrico do Rio de Janeiro das primeiras décadas do século XX. Observamos a existência inicial de críticas à categoria esquizofrenia, diante do uso hegemônico do diagnóstico de demência precoce, operadas por diferentes médicos psiquiatras, tanto nos textos científicos quanto na prática assistencial. Destacamos que foi no final da década de 1920 que o diagnóstico de esquizofrenia circulou de modo significativo em importantes instituições científicas e assistenciais no Rio de Janeiro, em detrimento do uso de demência precoce, por intermédio de apresentações em congresso, uso do novo diagnóstico em prontuários médicos, discussões clínicas em sociedade científica, artigos especializados e teses. Ou seja, tanto o Pavilhão de Observação do Hospício Nacional de Alienados quanto a Sociedade Brasileira de Psiquiatria, Neurologia e Medicina Legal, a partir de suas reuniões, foram coevos no movimento de apropriação da categoria esquizofrenia que então circulava nos espaços institucionais franceses, germânicos e espanhóis.

Demonstramos, ademais, que a incorporação da categoria esquizofrenia nesses espaços valeu-se do diálogo, presente no contexto europeu, entre as ideias psicodinâmicas de Bleuler e a teoria constitucionalista de Kretschmer, com referências a esses autores e a outros que adotavam a citação cruzada a ambos, as quais circularam em terras brasileiras tanto por meio da leitura e citação de originais quanto por meio de tradução e apropriação em francês e castelhano. Sublinhamos, assim, a relevância desse circuito linguístico plural expresso nos episódios de circulação e apropriação do campo semântico “esquizo” na psiquiatria brasileira no início do século XX. Como ocorrera nos espaços institucionais franceses, germânicos e espanhóis, os psiquiatras brasileiros participaram ativamente da circulação transnacional da categoria esquizofrenia por meio de sua apropriação e pelo uso mais ampliado do campo semântico “esquizo” consoante a interesses locais.

## NOTAS

1 Estamos nos referindo tanto a livros clássicos de história da psiquiatria que constroem uma história *whig* (Alexander, Selesnick, 1980), os relativos à história crítica da clínica (Bercherie, 1980; Noll, 2011), assim como a análises de autores ibero-americanos sobre o diagnóstico de esquizofrenia (Novella, Huertas, 2010; Conti, 2010; Álvarez, Colina, 2011; Golcman, 2015).

2 O periódico *Arquivos Brasileiros de Neuriatria e Psiquiatria* foi editado pela primeira vez em 1905, com o nome de *Arquivos Brasileiros de Psiquiatria, Neurologia e Ciências Afins*. Em 1907, é renomeado *Arquivos Brasileiros de Psiquiatria, Neurologia e Medicina Legal*, passando a ser vinculado à sociedade científica que levava o mesmo nome e era inaugurada naquele ano. Em 1919, a publicação passa a ser intitulada *Arquivos Brasileiros de Neuriatria e Psiquiatria.*

3 O Hospício Nacional de Alienados foi a primeira instituição assistencial psiquiátrica especializada no Brasil, fundada em 1852, no período imperial com o nome de Hospício de Pedro II e remodelada desde o início do contexto republicano, ganhando a nova denominação. Em 1911, passou a ser chamada Hospital Nacional de Alienados. É a instituição psiquiátrica mais pesquisada pela historiografia nacional, sendo uma das obras mais recentes a seu respeito a coletânea de Venancio e Dias (2022).

4 No Brasil, Henrique Roxo, Murillo de Campos, Heitor Carpinteiro Péres e Augusto Luís Nobre de Mello, membros da Sociedade Brasileira de Neurologia, Psiquiatria e Medicina Legal, leram e discutiram trabalhos de Minkowski.

5 Sobre a história desse pavilhão e suas relações com o Hospício Nacional de Alienados, ver Venancio (2003) e Mathias (2017, 2022).

6 O levantamento quantitativo aqui apresentado foi realizado por Carolina da Fonseca Schlaepfer, bolsista de iniciação cientifica do Pibic/CNPq-Fiocruz (2018-2020) sob orientação de Ana Teresa A. Venancio. A documentação consultada se constituiu dos livros-índices dos pacientes internados no Pavilhão de Observação do HNA.

7 Cabe destacar que o número de entradas não expressa exatamente o número de pacientes internados no HNA, tendo em vista que pacientes que passavam pelo Pavilhão de Observação poderiam não ser dirigidos depois para uma internação mais prolongada no HNA. Além disso, ao longo do período analisado, um mesmo paciente pode ter dado entrada mais de uma vez no Pavilhão de Observação.
